# Only half of the mothers practiced early initiation of breastfeeding in Northwest Ethiopia, 2015

**DOI:** 10.1186/s13104-017-2823-2

**Published:** 2017-10-10

**Authors:** Amare Tariku, Gashaw Andargie Biks, Molla Mesele Wassie, Abebaw Gebeyehu Worku, Melaku Kindie Yenit

**Affiliations:** 1 Department of Human Nutrition, Institute of Public Health, College of Medicine and Health Sciences, University of Gondar, Gondar, Ethiopia; 2 Department of Health Service Management and Health Economics, Institute of Public Health, College of Medicine and Health Sciences, University of Gondar, Gondar, Ethiopia; 3 Department of Reproductive and Child Health, Institute of Public Health, College of Medicine and Health Sciences, University of Gondar, Gondar, Ethiopia; 4 Department of Epidemiology and Biostatistics, Institute of Public Health, College of Medicine and Health Sciences, University of Gondar, Gondar, Ethiopia

**Keywords:** Early initiation of breastfeeding, Children aged 6–24 months, Health and Demographic Surveillance System, Ethiopia

## Abstract

**Background:**

Early initiation of breastfeeding has been well-recognized in reducing neonatal mortality; however, it remains sub-optimal in Ethiopia. This study therefore assessed the prevalence of early initiation of breastfeeding and associated factors among mothers with children aged 6–24 months in Dabat Health and Demographic Surveillance System (HDSS) site, northwest Ethiopia, where literature on the issue is markedly scarce.

**Methods:**

This community-based cross-sectional survey was carried out from May to June, 2015, at Dabat HDSS site, Dabat District. Eight hundred twenty-two mother–child pairs were included in the study. A multivariable logistic regression model was employed to identify factors associated with early initiation of breastfeeding.

**Results:**

This study demonstrated that the prevalence of early initiation of breastfeeding was 53.3%. Institutional delivery (AOR = 4.9; 95% CI 3.2, 7.4), higher Infant and Young Child Feeding (IYCF) knowledge (AOR = 2.3; 95% CI 1.6, 3.3), higher wealth status (AOR = 4.1, 95% CI 2.8, 6.0) and low fathers’ education (AOR = 0.3, 95% CI 0.2, 0.6) were significantly associated with early initiation of breastfeeding in the multivariate analysis.

**Conclusion:**

In summary, the coverage of early initiation of breastfeeding in Dabat HDSS site was low, considerably below the national target. Therefore, efforts should be intensified to step-up early initiation of breastfeeding by focusing on the identified determinants.

## Background

Breastfeeding is a well-recognized child survival strategy [1] as it has been proved in reducing the risk of different childhood diseases, including diarrhea and acute respiratory tract infections [1–4]. Particularly, a significant reduction in neonatal mortality is documented with initiation of breastfeeding within 1 h after birth, commonly named Early Initiation of Breastfeeding (EIBF) [1, 3, 5–8]. On the other hand, EIBF improves lactation and uterine contraction following delivery [7]. In spite of these priceless benefits, EIBF is sub-optimally practiced in many countries of the world [4]. EIBF practice ranges from 22 to 63% in Africa, [9–12], while it is 42.2–83.3% in Asia [13, 14]. Surprisingly, only one in ten (11.4%) mothers practiced EIBF in Saudi Arabia [15]. The coverage of EIBF is 35.2 and 47.1% in Turkey and Brazil, respectively [16, 17].

Likewise, the prevalence of EIBF is found low in Ethiopia; nationally 52% of the mothers practice EIBF [7]. Some of the district level studies also illustrate low prevalence of EIBF as it was shown in Jimma Arijo (62.6%) [18], Goba (52.4%) [19], Debre Markos (51.8%) [20] and Arbaminch (42.8%) [21]. However, majority of mothers initiated breastfeeding early in Nekemte District (88.5%) [22] and East Wollega Zone (83.3%) [23].

Based on the findings of former researches high EIBF practice was noted among mothers who had better infant and young child feeding (IYFC) knowledge [18, 20, 23–27], antenatal care (ANC) follow up, got breastfeeding counseling [9, 16, 24], and gave birth at health facilities [9, 12, 14, 28, 29]. Improved mothers’ education [10, 18, 19, 21, 30] and socioeconomic status [9, 24, 30, 31] also increases the liklihood of EIBF practice.

Nationally, different strategies have been implemented in Ethiopia to enhance infant and child feeding practice, including EIBF [32–34], nevertheless most of the infants still feed sub-optimally [7]. In addition, researches are limited. Therefore, to fill the knowledge gap, this study investigated EIBF and associated factors among mothers who had children aged 6–24 months at Dabat Health and Demographic Surveillance System (HDSS) site.

## Methods

This cross-sectional survey was carried out from May to June 2015 in Dabat HDSS site situated in Dabat District, Ethiopia. This surveillance site was instituted in 1996 and currently it expands its scope of investigation by including 13 kebeles (smallest administrative units). A total of 67,385 people are living in the site. The details of information about the surveillance site is available elsewhere [35, 36].

This study is part of the big survey entitled ‘Child Nutritional Status and Feeding Practice’. All children (6–59 months) with their mothers living in the HDSS site were participated in above survey. Representative eight kebeles were chosen through lottery method, then all mother–child pairs living in these kebeles were included. However, mothers with less than 6 months of residency were excluded from the survey. Epi-info version 3.7 was used to estimate a sample size of 804 by considering the expected proportion of mothers practicing EIBF as 52% [7], 95% confidence level, 5% margin of error, 5% non-response rate and a design effect of 2. However, we included all 822 mother–child pairs fulfilling the criteria for analysis to enhance the power of the study.

A structured and pretested questionnaire was used to collect data. Seventeen research assistants (14 data collectors and 3 supervisors) were recruited for the survey. English version questionnaire was translated to Amharic, the native language of the study area, and retranslated to English. Research assistants were trained and the tool was piloted before the actual data collection. EIBF, the outcome variable, is defined as putting the newborn to the breast within 1 hour of birth [1]. However, most of the mothers reported the time at which the infant received breast milk rather than telling the time at which they first put the newborn to the breast because they misunderstood the question [37]. Therefore, the study participants were asked how long after birth they first put [name] to the breast even if their breast milk did not arrive early enough.

Concerning the measurement of explanatory variables, household wealth status and mother’s infant and young child feeding knowledge were estimated using principal component analysis (PCA). For both variables, factor scores were summed and ranked into terciles (poor, medium and high). Further information about the items and approaches used for dependent variables ascertainment is available from the previous publication [35].

EPI-Info version 3.5.3 was used for data entry, while Statistical Package for Social Sciences (SPSS) version 20 analysis used for analysis. Hence clustering effect was not detected among kebeles of the HDSS site, individual level analysis was carried out. A binary logistic regression model was fitted to identify factors associated with EIBF. Initially bivariable analysis was done to show the independent effect of each explanatory variable on the outcome variable, EIBF. A variable selection criteria of p values of < 0.2 in the bivariable analysis was used for the final model, consequently all variables fulfilling the criteria (mothers and fathers’ education and employment status, mothers IYCF knowledge, place of delivery, health care access and household wealth status) were fitted to the multivariable logistic regression model. Significance of association was declared at a p value of < 0.05. The strength of association was shown using crude odds ratio (COR) and adjusted odds ratio (AOR) with a 95% confidence intervals. Multi-collinearity was checked, considering the variance inflation factor (VIF), none of the independent variables were co-linear. The Hosmer and Lemeshow goodness of fit-test was estimated at 0.74, suggesting the model fitted the data well.

## Results

The mean (± Standard Deviation, SD) age of children was 17.3 (± 7.3) months. Nearly three-quarters (67.4% and 69.5%, respectively) of the fathers and mothers were illiterate (Table 1). About 37.0% of participants had no history of ANC follow up and 75.9% gave birth at home (Table 2). This study demonstrated that the prevalence of EIBF was 53.3% [95% CI: 49.8, 56.7]. Moreover, the result of multivariate logistic regression analysis illustrated that place of delivery, mother’s IYCF knowledge, household wealth status, and father’s educational status were significantly and independently associated with EIBF.

**Table 1:** Socio-demographic and economic characteristics of the study participants in Dabat HDSS site, Dabat District, northwest Ethiopia, 2015

Variables	Frequency	Percent
Child age (months)		
6–11	239	29.1
12–24	583	70.9
Child sex		
Male	410	49.9
Female	412	50.1
Religion		
Orthodox	773	94.0
Other^a^	49	6.0
Mother’s age		
15–19	46	5.6
20–34	461	56.1
≥ 35	315	38.3
Mother’s education		
Illiterate	571	69.5
Literate	251	30.5
Mother’s employment		
Housewife	482	58.6
Farmer	211	25.7
Other^b^	129	15.7
Mother’s marital status		
Currently married	739	89.9
Currently unmarried^c^	83	10.1
Father’s education		
Illiterate	554	67.4
Primary school	143	17.4
Secondary school and above	125	15.2
Father’s employment		
Unemployed	46	5.6
Farmer	431	52.4
Other employment^d^	345	42.0
Household size		
≤ 4	304	37.0
> 4	518	63.0
Number of children under 5 years		
1	54	6.6
2–3	768	93.4
Wealth status		
Poor	307	37.3
Medium	246	29.9
High	269	32.7

^a^Muslim, protestant and catholic

^b^Students, unemployed, servant, own business

^c^Single, divorced and widowed

^d^Merchant, contract and permanent work

**Table 2:** Maternal health care utilization in Dabat HDSS site, Dabat District, northwest Ethiopia, 2015

Variables	Frequency	Percent
Antenatal care visits		
No visit	304	37.0
1	54	6.6
2–3	286	34.8
≥ 4	178	21.6
Place of delivery		
Home	624	75.9
Health facilities	198	24.1
Delivery attendant		
Health professionals	203	24.7
Traditional birth attendant	153	18.6
Relatives and volunteers	466	56.7
Postnatal follow-up		
Yes	212	25.8
No	610	74.2
Health care access		
Good access	607	73.8
Poor access	215	26.2
Health extension visit in the past 6 months		
No visit	391	47.5
1–2 visit	327	39.8
≥ 3	104	12.7
Mother’s IYCF^a^ knowledge		
Poor	272	33.1
Medium	285	34.7
High	268	32.2
Prelacteal feeding given to the child		
Yes	220	26.8
No	602	73.2

^a^Infant and Young Child Feeding

Accordingly, higher odds of EIBF were noted among mothers who gave birth at a health facility [AOR = 4.9; 95% CI 3.2, 7.4] compared to those who did it at home. Mothers with medium [AOR = 2.1; 95%CI 1.4, 3.1] and high [AOR: 2.3; 95% CI 1.6, 3.3] IYCF knowledge had increased odds of EIBF compared to mothers with poor IYCF knowledge. Likewise, the odds of EIBF were higher among mothers in medium [AOR = 2.4, 95% CI 1.6, 3.6] and high wealth status categories [AOR = 4.1, 95% CI 2.8, 6.0] compared to those who were in poor wealth status category. However, the odds of EIBF were lower among children whose fathers were either illiterate [AOR = 0.3, 95% CI 0.2, 0.6] or attended primary school [AOR = 0.5, 95% CI 0.3, 0.9] than children whose fathers attended secondary school and above (Table 3).

**Table 3:** Factors associated with early initiation of breastfeeding among mothers who had children aged 6–24 months in Dabat HDSS site, Dabat District, northwest Ethiopia, 2015

Variables	Early initiation of breastfeeding		Crude odds ratio (95%CI^**^)	Adjusted odds ratio (95%CI)
	Yes (#)	No (#)		
Mother’s education				
Illiterate	270	301	1	1
Literate	114	137	1.1 (0.8, 1.5)	1.1 (0.7, 1.3)
Mother’s employment				
Farmer	84	127	1.4 (0.9, 2.0)	0.8 (0.4, 1.5)
Housewife	272	210	1	1
Others	82	47	0.8 (0.4, 1.5)	1.4 (0.9, 2.0)
Father’s employment				
Unemployed	31	15	2.1 (1.1, 3.9)	1.6 (0.7, 3.3)
Farmer	234	197	1.2 (0.9, 1.6)	1.3 (0.9, 1.8)
Others	173	172	1	1
Father’s education				
Illiterate	286	268	0.2 (0.1, 0.4)	0.3 (0.2, 0.6)*
Primary school	57	86	0.3 (0.2, 0.5)	0.5 (0.3, 0.9)*
Secondary school and above	95	30	1	1
Wealth status				
Poor	103	204	1	*1*
Medium	172	97	3.5 (2.5, 4.6)*	2.4 (1.6, 3.6)*
High	163	83	3.9 (2.7, 5.6)*	4.1 (2.8, 6.0)*
Mother’s IYFC knowledge				
Poor	114	158	1	*1*
Medium	155	110	2.0 (1.4, 2.8)*	2.1 (1.4, 3.1)*
High	169	116	2.0 (1.4, 2.8)*	2.3 (1.6, 3.3)*
Place of delivery				
Home	283	341	1	*1*
Health facility	155	43	4.4 (3.0, 6.3)*	4.9 (3.2, 7.4)^*^
Health care access				
Good access	313	294	0.8 (0.6, 1.1)	0.9 (0.6, 1.4)
Poor access	125	90	1	1

* Significant at a p value of < 0.05

** Confidence interval

## Discussion

In the Health Sector Development Programme IV, Ethiopia set a target to raise EIBF practice to 92% by the end of 2015 [32]. However, this study demonstrated that only 53.5% of mothers initiated breastfeeding within one hour of birth. The finding was consistent with the 2011 Ethiopian Demographic Health Survey (DHS) report (52%) [7] and other local studies done in Arbaminch and Goba districts which reported 57.2% [21] and 52.4% [19], respectively.

However, our finding was the lowest compared to findings from other parts of Ethiopia, for example, Nekemtie District (88.5%) [22], East Wollega Zone, (83.5%) [23], and Jimma Arjo District (63%) [18]. The low prevalence of EIBF in the current study could be related to the lower utilization of institutional delivery compared to the studies done in Nekemtie District and East Wollega Zone. Other Previous reports claimed that institutional delivery was associated with a higher likelihood of EIBF [9, 14, 28]. On the other hand, different studies documented that prelacteal feeding was higher among mothers who gave birth at home [35, 38] and this is associated with lower odds of EIBF [38, 39].

Findings similar to ours, for example, 46.1% [11, 12], 56% [9], and 47.1% [16] were reported from Tanzania, Uganda, and Brazil, respectively. On the other hand, the prevalence of EIBF in our work was higher than the reports from Saudi Arabia (11.4%) [15], India (22%) [40], Bangladesh (46.3%) [13], Nepal (42.2%) [24], and Turkey (35.2%) [17]. Maternal health care utilization and access to child feeding information were higher in the latter study areas [15, 40]. Therefore, a lower prevalence of EIBF in the above overseas studies could be related to mothers’ perception of the inadequate and delayed production of breast milk which forced them to give prelacteal feeds. In fact, mother’s unfavorable perception of breastfeeding was related to delayed initiation and subsequent sub-optimal breastfeeding practices [41, 42].

The result of the adjusted analysis indicated that place of delivery, mother’s IYCF knowledge, household wealth status, and fathers’ education were significantly and independently associated with EIBF, while household wealth status was positively associated with it. The finding was supported by reports from some developing countries [9, 24, 30, 31]. This could be partially explained by the positive effect of socioeconomic status on mothers’ health care utilization, institutional delivery, for instance. The Ethiopian DHS report (2011) also showed that mothers’ institutional delivery preference was enhanced with the improvement of household socio-economic status [7].

Similarly, this study showed that the odds of EIBF were high among mothers who gave birth at health facilities. This was in agreement with the reports from other African and Asian countries [9, 12, 14, 28, 29]. Obviously, institutional delivery creates a chance to deliver skilled guidance/support about appropriate neonatal feeding information and serves as the only way to deliver immediate obstetric cares, including supporting mothers to initiate breastfeeding imminently after birth. Breastfeeding support and counseling by health care professionals is one of the crucial interventions to step-up early initiation into breastfeeding [43–46]. However, 43 (21.7%) of the mothers who gave birth at health facilities delayed the initiation into breastfeeding. This could be related to the poor commitment of health care professionals to support mothers in practicing early initiation into breastfeeding. This however requires further investigations.

A lower paternal educational status (illiterate and primary school) was associated with decreased odds of EIBF compared to those who had higher educational status, secondary school and above. The finding was supported by studies done elsewhere [19, 21, 24, 28]. In fact, paternal support and favorable attitude were affirmed in boosting mothers’ confidence to practice optimal breastfeeding [42, 47, 48]. Also, education is a crucial tool in enhancing parent’s awareness of appropriate neonatal feeding practices [7]. In line with earlier findings [20, 23, 24, 26, 27, 49], this study illustrated that the odds of EIBF among mothers with higher IYCF knowledge were higher compared to those who had low IYCF knowledge. Stepping-up mothers IYCF knowledge is one of the key interventions to mitigate the high burden of inappropriate infant and child feeding practices [34]. Despite its importance, most of the national nutrition strategies which are designed to improve IYCF practice, did not adequately describe behavioral change and communication components [33, 34].

Our study showed the extent and factors associated with EIBF practice in the rural population of northwest Ethiopia where health care and other social services, including education, are not adequacy accessible. However, some limitation should be understood. Though efforts were made (as mentioned in the method section) to help the mother to remember the actual time of initiation of breastfeeding, the study is not free from recall bias. Hence, this study utilized a data collected to answer another research question; some of the other variables, such as birth weight were not included. Given that, selection bias is the other possible limitation of the study.

## Conclusion

In summary, despite the fact that there are implementations of programs and strategies in Dabat HDSS site, the coverage of EIBF was considerably below the national target. The result also suggested that EIBF was associated with health care utilization and socio-economic factors. This implies that further improvements of the utilization of institutional delivery and paternal involvement are critical to enhance EIBF coverage. Also, increasing mothers’ IYCF knowledge through strengthening breastfeeding education and counseling programs are essential. Finally, a longitudinal study approach is highly recommended for further investigations.

## Abbreviations

HDSS: Health and Demographic Surveillance System
AOR: adjusted odds ratio
COR: crude odds ratio
CI: confidence interval
IYCF: infant and young child feeding
EIBF: early initiation of breastfeeding
WHO: World Health Organization
ANC: antenatal care
SPSS: Statistical Package for Social Sciences
SD: standard deviation
